# The Heparan Sulfate Sulfotransferases HS2ST1 and HS3ST2 Are Novel Regulators of Breast Cancer Stem-Cell Properties

**DOI:** 10.3389/fcell.2020.559554

**Published:** 2020-09-25

**Authors:** Felipe C. O. B. Teixeira, Archana Vijaya Kumar, Sampath Kumar Katakam, Cinzia Cocola, Paride Pelucchi, Monika Graf, Ludwig Kiesel, Rolland Reinbold, Mauro S. G. Pavão, Burkhard Greve, Martin Götte

**Affiliations:** ^1^Instituto de Bioquímica Médica Leopoldo de Meis, Hospital Universitário Clementino Fraga Filho, Universidade Federal do Rio de Janeiro, Rio de Janeiro, Brazil; ^2^Department of Gynecology and Obstetrics, Münster University Hospital, Münster, Germany; ^3^Istituto di Tecnologie Biomediche, Consiglio Nazionale delle Ricerche, Segrate, Italy; ^4^Department of Radiotherapy and Radiooncology, University Hospital of Münster, Münster, Germany

**Keywords:** breast cancer, sulfotransferase, heparan sulfate, epithelial-to-mesenchymal transition, cancer stem cell (CSC), syndecan, notch, Sulf1

## Abstract

Heparan sulfate (HS) is a glycosaminoglycan found mainly in its protein-conjugated form at the cell surface and the extracellular matrix. Its high sulfation degree mediates functional interactions with positively charged amino acids in proteins. 2-O sulfation of iduronic acid and 3-O sulfation of glucosamine in HS are mediated by the sulfotransferases HS2ST and HS3ST, respectively, which are dysregulated in several cancers. Both sulfotransferases regulate breast cancer cell viability and invasion, but their role in cancer stem cells (CSCs) is unknown. Breast CSCs express characteristic markers such as CD44^+^/CD24^−/*low*^, CD133 and ALDH1 and are involved in tumor initiation, formation, and recurrence. We studied the influence of HS2ST1 and HS3ST2 overexpression on the CSC phenotype in breast cancer cell lines representative of the triple-negative (MDA-MB-231) and hormone-receptor positive subtype (MCF-7). The CD44^+^/CD24^−/*low*^ phenotype was significantly reduced in MDA-MB-231 cells after overexpression of both enzymes, remaining unaltered in MCF-7 cells. ALDH1 activity was increased after HS2ST1 and HS3ST2 overexpression in MDA-MB-231 cells and reduced after HS2ST1 overexpression in MCF-7 cells. Colony and spheroid formation were increased after HS2ST1 and HS3ST2 overexpression in MCF-7 cells. Moreover, MDA-MB-231 cells overexpressing HS2ST1 formed more colonies and could not generate spheres. The phenotypic changes were associated with complex changes in the expression of the stemness-associated notch and Wnt-signaling pathways constituents, syndecans, heparanase and Sulf1. The results improve our understanding of breast CSC function and mark a subtype-specific impact of HS modifications on the CSC phenotype of triple-negative and hormone receptor positive breast cancer model cell lines.

## Introduction

Heparan sulfate (HS) is a highly sulfated glycosaminoglycan (GAG) found on the cell surface and in the extracellular matrix (ECM) (Zhang, 2010; Karamanos et al., 2018). Its localization is determined by the core protein, while the HS chains determine the affinity to numerous ligands such as growth factors, cytokines, proteases, lipoproteins and ECM components (Denys and Allain, 2019). HS mediates cell-cell and cell-ECM communication, leading to different pathological and physiological effects, including embryonic development, cell growth and differentiation, homeostasis, inflammatory responses, tumor growth and microbial infection (Li and Kusche-Gullberg, 2016). These interactions are driven by the high degree of sulfation of this molecule, which interacts with positively charged amino acid residues in the protein ligands (Morla, 2019). The spatial arrangement of sulfate groups in different HS domains is equally crucial to ensure optimal binding to different ligands (Esko and Selleck, 2002). The high structural variability of HS stems from enzymatic modifications of its glycan backbone of repeated disaccharide units of D-glucuronic acid (GlcA) and *N*-acetyl-D-glucosamine (GlcNAc) synthesized in the Golgi apparatus. Sequential modification steps include deacetylation, epimerization and, finally, sulfation catalyzed by the enzymes *N*-deacetylases/*N*-sulfotransferases (NDSTs) and 2-O, 6-O and 3-O sulfotransferases (HS2ST, HS6ST, and HS3ST, respectively) (Li and Kusche-Gullberg, 2016). While 2-O sulfation occurs at the uronic acids (mainly IdoA), 6-O and 3-O sulfation occur at the GlcN residues (Zhang, 2010). Aberrant regulation of sulfotransferase expression affects several processes regulated by HS, ranging from organ development to inflammation (Merry and Wilson, 2002; Denys and Allain, 2019).

The 2-O sulfated iduronic acid (IdoA2S) is a widely common HS motif and mediates the binding and signaling of several growth factors, whereas 3-O-sulfation of glucosamine is the rarest HS modification step, yet strongly mediating selective cellular processes (Kreuger et al., 2001; Witt et al., 2013). In humans, seven HS3STs and one HS2ST have been characterized, and its expression depends on the cell type and tissue environment (Gulberti et al., 2020). Notably, HS3ST is misexpressed in different types of cancers. However, its effect on cancer is still not clear, as some reports show antitumoral effects and others describe tumor-promoting activities (Vijaya Kumar et al., 2014; Denys and Allain, 2019).

Cancer stem cells (CSC), represent a population of cancer cells within a tumor responsible for tumor initiation, formation, and recurrence (Eun et al., 2017; Vitale et al., 2019). Breast cancer stem cells (BCSC) have a number of markers, such as CD44, CD24, aldehyde dehydrogenase 1 (ALDH1), among others.

CD44 is a transmembrane glycoprotein that acts as a receptor for hyaluronic acid and its expression in CSCs is associated with a mesenchymal phenotype, associated with increased adhesion, invasion and cell migration (Jaggupilli and Elkord, 2012; Li et al., 2017; Vitale et al., 2019). On the other hand, CD24 is associated with carbohydrate metabolism and a more epithelial phenotype in breast cancer (Park et al., 2010). CSCs that have the CD44^+^/CD24^–^ phenotype, therefore, are associated with a more mesenchymal and highly metastatic/invasive phenotype with greater tumorigenic potential (Li et al., 2017). Expression of ALDH1, an intracellular enzyme that oxidizes aldehydes and retinol in CSCs, is associated with an epithelial phenotype and has been shown to lead to treatment resistance, a more aggressive phenotype, and a worse prognosis on patients (Liu et al., 2014; Saeg and Anbalagan, 2018).

HS2ST1 expression is dysregulated in several tumor entities, suggesting a role in tumor progression (Bonuccelli et al., 2009; Zhao and Wang, 2020). Dysregulated expression of HS2ST1 is associated with a CSC and metastasis-associated signature in breast cancer cells carrying a mutation in caveolin (Bonuccelli et al., 2009), and upregulation of HS2ST1 is associated with reduced invasive behavior and senescence in breast cancer cells (Kang et al., 2020; Vijaya Kumar et al., 2020). HS3ST2 overexpression, on the other hand, has breast cancer cell-type-dependent effects on invasion and proliferation, affects stemness-associated signaling pathways and protects breast cancer cells against apoptosis and natural killer cell-mediated cell death (Vijaya Kumar et al., 2014; Hellec et al., 2018). In this scenario, we aimed to elucidate the influence of HS2ST1 and HS3ST2 HS sulfotransferases on the acquisition of a CSC phenotype and the expression of BCSC markers in two distinct breast cancer cell lines, MDA-MB-231 (triple negative, mesenchymal phenotype) and MCF-7 (ER+/ PR+/HER2−, epitheloid morphology).

## Materials and Methods

### Cell Lines and Reagents

MDA-MB-231 and MCF-7 cells were from ATCC/LGC Promochem (Wesel, Germany) and stably transfected with a pcDNA3.1 control plasmid (Invitrogen, Karlsruhe, Germany) or a plasmid allowing for expression of the open reading frame (1104 bp) of human HS2ST1 (NCBI Reference Sequence: NM_012262) or human HS3ST2 (NCBI Reference Sequence: NM_006043.1) in the vector pReceiver-M02 under control of the cytomegalovirus promoter (RZPD/ImaGenes, Berlin, Germany) as previously described (Nikolova et al., 2009; Vijaya Kumar et al., 2014, 2020). Stable clones were selected using 1 mg/ml G418. MDA-MB-231 cells were maintained in Dulbecco’s Modified Eagle Medium – High Glucose (DMEM-HG) containing 10% fetal calf serum (FCS), 1% penicillin/streptomycin and 600 mg/ml G418 in a humidified atmosphere of 7% CO_2_ at 37°C. Successful transfection was confirmed by qPCR (Vijaya Kumar et al., 2014, 2020). MCF-7 cells were cultured in RPMI-1640 medium containing 10% FCS, 1% penicillin/streptomycin and 600 mg/ml G418 in a humidified atmosphere of 5% CO_2_ at 37°C. Media, FCS and tissue culture supplies were from Gibco BRL (Karlsruhe, Germany). siRNA knockdown of Syndecan-1 and Syndecan-4 in some experiments was done as previously described (Ibrahim et al., 2012) using siRNAs #s12634 (Sdc1), # s12638 (Sdc4) and a negative control siRNA (negative control #1; all from Ambion, Cambridgeshire, United Kingdom). Unless stated otherwise, all chemicals were from Sigma (Deisenhofen, Germany). In some experiments, cells were treated with 1 μM gamma secretase inhibitor (GSI, Calbiochem, Darmstadt, Germany) for 24 h as previously described (Ramirez Williams et al., 2019).

### CD24 and CD44 Identification With Flow Cytometry

To detect CD24 and CD44, cells were incubated with 10 μl of anti-human-CD44-FITC and anti-human-CD24-PE or the IgG2b-FITC and IgG1-PE isotype control antibodies (Immunotools, Friesoythe, Germany) for 30 min at room temperature in the dark. Stained cells were analyzed by a Cyflow Space flow cytometer (Sysmex/Partec, Münster, Germany).

### Identification of ALDH-1 Positive Cells

For ALDH1 activity assessment, we used the ALDEFLUOR^TM^ kit (StemCell Technologies, Köln, Germany) as previously described (Ibrahim et al., 2013). Briefly, 2 × 10^5^ MDA-MB-231 and MCF-7 control cells or overexpressing HS2ST1 and HS3ST2 were resuspended in assay buffer containing ALDH1 substrate (1 μmol/L). Another pool of control cells were incubated with 50 mM ALDH1 inhibitor diethylaminobenzaldehyde (DEAB) as negative control. These solutions of cells were incubated for 1 h at 37°C in a water bath in the dark with agitation at 10 min interval. After 1 h, the cells were centrifuged at 400G for 5 min and resuspended in 1 mL assay buffer and stored on ice prior to acquisition with a Cyflow Space cytometer.

### Colony Formation Assay

To examine the effect of the overexpression of HS2ST1 and HS3ST2 on colony formation, 800 control and transfected cells were seeded in 35 mm gridded dishes and maintained in their respective media with 10% FCS for 10–14 days. The total number of colonies was counted using a microscope and the percentage of altered colony numbers was accessed as a ratio between sulfotransferase overexpressing cells and vector control cells.

### Analysis of Spheroid Size by the Hanging Drop Method

To access the spheroid formation ability and compare the spheroid size of the cells, we first prepared a solution of 10^6^ cells/mL in complete medium and placed several 20 μl drops into the lid of a Petri dish, after which we added 7 mL sterile PBS to the bottom of the dish and left it in the cell incubator for 1 week. Pictures of the spheroids inside the drop were taken using a Zeiss Axiophot camera and their comparative size was obtained measuring the area occupied by the spheres using the software NIH ImageJ (NIH, Bethesda, United States).

### Mammosphere Assay

For the mammosphere formation assay, a culture medium containing DMEM/F12 supplemented with 2 mM L-glutamine and 100 U/mL penicillin/streptomycin was prepared. Immediately before use, 20 ng/ml of recombinant human epidermal growth factor (EGF) and 10 ng/ml of basic human fibroblast growth factor (bFGF) were added. Cells were detached from the flasks and resuspended in complete mammosphere medium. 2 × 10^3^ cells of each condition were added to the wells of an ultra-low adhesion 6-well plate (Cornind Costar, Darmstadt, Germany) and placed in a cell incubator at 37°C and 5% CO_2_ for 9 or 15 days. Subsequently, spheres were counted and the number of spheres per number of cells initially plated was calculated.

### Quantitative Real-Time PCR

The total RNA isolated from cultured cells using a kit (Analytik Jena, Jena, Germany) was reverse transcribed into cDNA using the high capacity cDNA kit (Applied Biosystems, Foster City, CA, United States). Quantitative real-time PCR was conducted in duplicate for each gene of interest using TaqMan probes or SYBR Green dye and gene expression levels were measured in a steponeplus detection system (Applied Biosystems). Relative gene expression was evaluated using the 2^–ΔΔ*Ct*^ method after normalization to 18S rRNA or beta-actin as previously described (Ibrahim et al., 2012). Primer information is provided in Supplementary Table I.

### Western Blotting

Western blotting was performed using 30 μg of cell extract/lane exactly as previously described (Ibrahim et al., 2012). Membranes were stripped and reprobed with tubulin antibodies as loading control. Antibodies are listed in Supplementary Table II.

### Statistical Analysis

All Data are presented as mean ± SEM or SD as indicated in the figure legends and mean ± SEM in the text. Biological replicates per independent experiments were as follows: Flow cytometry and colony formation (3 × 3), Mammosphere and hanging drop assay (3 × 10), qPCR (1–3 × 3–5). Western blot (2–4 × 2). Comparisons among two distinct groups were evaluated using Student’s *t*-test (for normally distributed data) or Mann–Whitney *U*-test (for non-normally distributed data). The statistical difference between more than two groups was evaluated by one-way ANOVA followed by Tukey’s multiple comparison test. The level of significance was set at *p* < 0.05. Graphs were plotted and analyses were performed by GraphPad Prism 7 software (San Diego, CA, United States).

## Results

### HS2ST1 and HS3ST2 Overexpression in MDA-MB-231 and MCF-7 Cells Alter the Expression of the CSC Markers CD24 and CD44, and ALDH1 Enzymatic Activity

**FIGURE 1:**
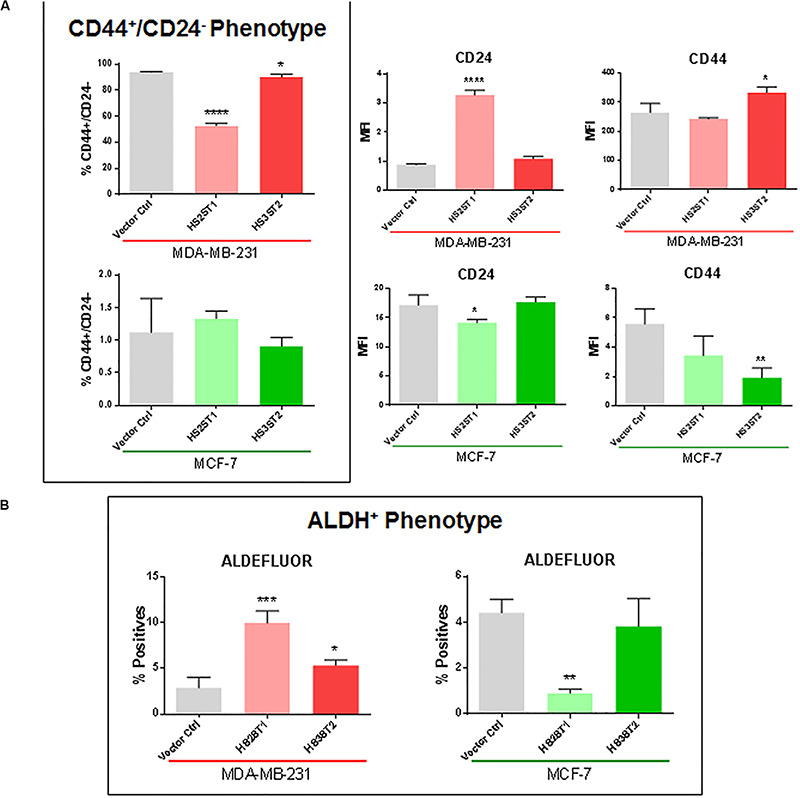
Expression of cancer stem cell markers CD24 and CD44 and ALDH1 enzyme activity. MDA-MB-231 and MCF-7 cells modified to overexpress HS2ST1 and HS3ST2 were labeled with anti-CD24 and anti-CD44 antibodies or incubated with the ALDEFLUOR^TM^ kit reagent and analyzed by flow cytometry. **(A, inside the box)** percentage of cells having the CD44^+^/CD24^–^ phenotype in the MDA-MB-231 and MCF-7 cells. **(A, outside the box)** Mean fluorescence intensity (MFI) of CD24 and CD44 in the MDA-MB-231 cells (top), or MCF-7 cells (bottom). **(B)** percentage of ALDH^+^ phenotype in the MDA-MB-231 and in the MCF-7 cells. Data represent mean ± SD, *n* = 3. *P < 0.05, **P < 0.01, ***P < 0.001, ****P < 0.0001.

First, we analyzed by flow cytometry whether the percentage of MDA-MB-231 and MCF-7 breast cancer cells displaying the CD44^+^/CD24^–^ phenotype was changed by HS2ST1 and HS3ST2 overexpression. The clones were already stablished and characterized by our group (Vijaya Kumar et al., 2014, 2020). qPCR revealed that HS2ST1 overexpression led to an 25-37-fold increase in HS2ST1 mRNA expression (Table 1), while we could only detect HS3ST2 mRNA in cells transfected with a HS3ST2 expression plasmid (Vijaya Kumar et al., 2014). In triple-negative MDA-MB-231 cells, upregulation of both sulfotransferases led to a significant decrease in the percentage of cells with the CD44^+^/CD24^–^ phenotype in comparison to the vector control cells (Figure 1A, highlighted in the box). HS2ST1 overexpression reduced this phenotype from 94.05% (±0.24%) in control cells to 52.83% (±1.06%) in the transfected cells, whilst cells overexpressing the HS3ST2 sulfotransferase presented 90.2% (±1.16%) of cells with the CD44^+^/CD24^–^ phenotype. In contrast, hormone-receptor positive MCF-7 cells did not undergo a significant change in this CD44^+^/CD24^–^ phenotype after HS2ST1 or HS3ST2 overexpression (Figure 1A). The number of CD44^+^/CD24^+^ cells significantly increased in the MDA-MB-231 cells after overexpression of HS2ST1 and HS3ST2, respectively, from 5.82% (±0.24%) in the vector control cells to 47.07% (±1.06%) in the HS2ST1 overexpressing cells and 9.72% (±1.17%) in the cells overexpressing HS3ST2 (data not shown). In MCF-7 cells, the overexpression of HS3ST2 significantly decreased the double-positive phenotype from 37.91% (±1.06%) in the vector control cells to 12.71% (±0.98%) in the transfected cells. Compared to the vector control cells (MDA-MB-231: 0.88 ± 0.02; MCF-7: 17.1 ± 1.03), overexpression of the HS2ST1 enzyme led to a significant increase in CD24 expression on the membrane of MDA-MB-231 cells (3.28 ± 0.09) and a significant reduction of this marker in MCF-7 cells (14.12 ± 0.32), as determined by measuring the mean fluorescence intensity (MFI) (Figure 1A). Compared to the vector control cells (MDA-MB-231: 264.93 ± 17.91; MCF-7: 5.58 ± 0.59), HS3ST2 overexpression led to a significant increase of CD44 in MDA-MB-231 cells (333.61 ± 11.07) and a reduction of its expression in MCF-7 cells (1.91 ± 0.39) (Figure 1A).

**TABLE 1:** pPCR analysis of breast cancer cell lines overexpressing HS sulfotransferases.

Genes	MCF-7					MDA-MB-231				
	Control	HS2ST1	*P*	HS3ST2	*P*	Control	HS2ST1	*P*	HS3ST2	*P*
**HS related**										
**HS2ST1 (*n* ≥ 10)**	1.00 ± 0.07	**24.89 ± 16.56**	**<0.001**	1.06 ± 0.23	0.51	1.01 ± 0.11	**37.42 ± 18.47**	**<0.0001**	1.10 ± 0.18	0.15
HS3ST2 (*n* ≥ 6)*	–	–	n.a.	+++	n.a.	–	–	n.a.	+++	n.a.
**Sulf1 (*n* ≥ 8)**	1.00 ± 0.20	**15.46 ± 12.02**	**<0.05**	**7.52 ± 4.53**	**<0.01**	1.02 ± 0.23	**3.88 ± 1.59**	**<0.01**	**1.65 ± 0.51**	**<0.01**
**Sulf2 (*n* ≥ 8)**	1.01 ± 0.12	0.84 ± 0.19	0.06	**0.72 ± 0.24**	**<0.05**	1.08 ± 0.42	2.62 ± 2.66	0.15	1.87 ± 1.42	0.17
**HPSE (*n* ≥ 10)**	1.03 ± 0.28	**1.97 ± 0.91**	**<0.001**	**1.50 ± 0.92**	**<0.05**	1.02 ± 0.20	**0.57 ± 0.31**	**<0.0001**	1.02 ± 0.31	0.97
**Syndecans**										
Sdc-1 (*n* ≥ 6)	1.02 ± 0.21	1.03 ± 0.18	0.93	1.03 ± 0.24	0.91	1.03 ± 0.27	0.75 ± 0.54	0.28	0.75 ± 0.60	0.33
**Sdc-2 (*n* ≥ 6)**	1.03 ± 0.28	1.04 ± 0.19	0.92	0.84 ± 0.26	0.31	1.01 ± 0.10	**2.04 ± 0.67**	**<0.04**	1.19 ± 0.31	0.86
Sdc-3 (*n* ≥ 3)	1.00 ± 0.09	1.09 ± 0.18	0.53	0.84 ± 0.07	0.07	1.01 ± 0.13	1.14 ± 0.58	0.74	0.79 ± 0.05	0.09
**Sdc-4 (*n* ≥ 8)**	1.02 ± 0.23	**1.80 ± 0.66**	**<0.001**	**2.06 ± 0.68**	**<0.01**	1.01 ± 0.16	1.54 ± 0.72	0.07	1.25 ± 0.70	0.37
**EMT**										
***E*-Cadherin (*n* ≥ 9)**	1.01 ± 0.06	**0.79 ± 0.20**	**<0.01**	0.88 ± 0.37	0.28	1.00 ± 0.04	1.24 ± 0.85	0.38	1.43 ± 0.67	0.09
***N*-Cadherin (*n* ≥ 7)**	1.15 ± 0.60	1.00 ± 0.50	0.60	0.96 ± 0.41	0.48	1.01 ± 0.12	**1.23 ± 0.19**	**<0.05**	0.82 ± 0.25	0.09
β**-catenin (*n* ≥ 7)**	1.01 ± 0.18	**1.42 ± 0.33**	**<0.05**	1.21 ± 0.29	0.13	1.01 ± 0.12	0.81 ± 0.32	0.14	1.15 ± 0.46	0.42
**ZEB1 (*n* ≥ 8)**	1.08 ± 0.46	1.34 ± 0.77	0.39	1.42 ± 0.63	0.23	1.00 ± 0.07	1.12 ± 0.29	0.27	**0.80 ± 0.22**	**<0.05**
ZEB2 (*n* ≥ 7)	0.93 ± 0.36	2.74 ± 4.02	0.28	2.38 ± 2.21	0.17	1.01 ± 0.17	1.51 ± 0.77	0.11	1.23 ± 0.34	0.14
**Vimentin (*n* ≥ 9)**	1.01 ± 0.05	**0.62 ± 0.34**	**<0.01**	0.79 ± 0.45	0.13	1.00 ± 0.01	1.02 ± 0.08	0.42	**0.87 ± 0.06**	**<0.0001**
**Snail1 (*n* ≥ 9)**	1.02 ± 0.24	**0.58 ± 0.46**	**<0.001**	**0.90 ± 0.19**	**<0.001**	1.00 ± 0.08	**0.67 ± 0.21**	**<0.01**	**1.41 ± 0.41**	**<0.05**
**Snail2 (*n* ≥ 16)**	1.08 ± 0.33	**0.64 ± 0.32**	**<0.05**	0.87 ± 0.50	0.54	1.01 ± 0.13	**0.68 ± 0.22**	**<0.01**	**1.32 ± 0.34**	**<0.05**
**Twist (*n* ≥ 9)**	1.00 ± 0.06	**0.08 ± 0.19**	**<0.0001**	**0.11 ± 0.25**	**<0.0001**	1.03 ± 0.27	3.05 ± 3.81	0.15	2.56 ± 3.70	0.25
**Notch signaling**										
Notch-1 (*n* ≥ 5)	1.03 ± 0.27	1.09 ± 0.17	0.67	0.92 ± 0.29	0.57	1.06 ± 0.41	1.33 ± 1.18	0.51	0.98 ± 0.27	0.58
**Notch-2 (*n* ≥ 3)**	1.00 ± 0.01	5.78 ± 6.25	0.32	4.62 ± 4.44	0.29	1.09 ± 0.50	**3.19 ± 1.57**	**<0.001**	**6.81 ± 5.72**	**<0.05**
**Notch-3 (*n* ≥ 5)**	1.02 ± 0.23	**1.69 ± 0.10**	**<0.01**	**1.54 ± 0.27**	**<0.05**	1.13 ± 0.62	1.59 ± 1.17	0.31	2.56 ± 2.52	0.11
Notch-4** (*n* ≥ 5)	1.35 ± 1.19	0.39 ± 0.39	0.08	1.21 ± 1.40	0.84	0.98 ± 0.34	0.65 ± 0.44	0.09	0.86 ± 0.52	0.59
**Numb (*n* ≥ 12)**	1.00 ± 0.11	1.20 ± 0.33	0.06	**1.16 ± 0.18**	**<0.01**	1.02 ± 0.21	**1.74 ± 1.07**	**<0.05**	1.04 ± 0,41	0.87
**DLL1 (*n* ≥ 3)**	1.00 ± 0.09	0.93 ± 0.33	0.75	**1.27 ± 0.28**	**<0.05**	1.01 ± 0.08	**1.23 ± 0.14**	**<0.0001**	n.d.	n.d.
DLL3 (*n* ≥ 8)	1.07 ± 0.42	1.43 ± 2.21	0.66	0.88 ± 0.96	0.52	1.04 ± 0.32	0.94 ± 0.48	0.61	0.82 ± 0.45	0.25
**DLL4 (*n* ≥ 8)**	1.03 ± 0.28	0.83 ± 0.40	0.26	**0.70 ± 0.32**	**<0.05**	1.03 ± 0.28	**1.77 ± 0.72**	**<0.05**	0.93 ± 0.18	0.43
**Hes1 (*n* ≥ 9)**	1.04 ± 0.29	1.05 ± 0.41	0.69	**0.80 ± 0.31**	**0.05**	1.02 ± 0.24	**2.04 ± 1.18**	**<0.05**	**1.73 ± 0.73**	**<0.05**
**Hes2 (*n* ≥ 16)**	1.03 ± 0.25	**0.72 ± 0.18**	**<0.0001**	0.87 ± 0.40	0.18	1.03 ± 0.18	**1.88 ± 0.80**	**<0.0001**	1.01 ± 0.47	0.91
**Hey1 (*n* ≥ 11)**	1.02 ± 0.10	**1.30 ± 0.29**	**<0.001**	1.01 ± 0.38	0.94	1.00 ± 0.07	0.87 ± 0.30	0.17	1.01 ± 0.13	0.88
**Hey2 (*n* ≥ 17)**	1.15 ± 0.69	**5.58 ± 7.96**	**<0.05**	**3.77 ± 4.18**	***p* < 0.05**	1.07 ± 0.44	1.10 ± 0.71	0.86	**1.66 ± 1.08**	***p* < 0.05**
**Jag1 (*n* ≥ 15)**	1.10 ± 0.50	**2.60 ± 1.28**	**<0.001**	**2.41 ± 0.94**	**<0.001**	1.02 ± 0.23	**1.45 ± 0.57**	**<0.05**	1.18 ± 0.78	0.46
**Wnt signaling**										
**Wnt1 (*n* ≥ 8)**	–	–	n.a.	–	n.a.	1.01 ± 0.46	1.07 ± 0.65	0.82	**2.48 ± 1.11**	**<0.01**
Wnt3a (*n* ≥ 8)	1.01 ± 0.18	1.61 ± 1.42	0.20	1.83 ± 1.62	0.13	1.07 ± 0.42	1.18 ± 0.77	0.71	1.32 ± 0.90	0.48
**Wnt5a (*n* ≥ 8)**	1.38 ± 1.15	1.39 ± 0.68	0.99	1.58 ± 0.92	0.72	1.00 ± 0.04	**0.49 ± 0.11**	**<0.0001**	**0.29 ± 0.11**	**<0.0001**
**FZD7 (*n* ≥ 8)**	1.02 ± 0.24	1.12 ± 0.40	0.57	1.13 ± 0.58	0.66	1.00 ± 0.08	**1.19 ± 0.14**	**<0.01**	**1.73 ± 0.60**	**<0.05**
SFRP1 (*n* ≥ 8)	1.20 ± 0.74	1.40 ± 1.69	0.73	1.16 ± 0.81	0.90	1.03 ± 0.27	1.58 ± 0.66	0.06	0.89 ± 0.54	0.52
**TCF7L1 (*n* ≥ 8)**	1.09 ± 0.31	**0.65 ± 0.40**	**<0.001**	**0.58 ± 0.24**	**<0.001**	1.02 ± 0.23	**4.23 ± 3.10**	**<0.05**	**1.63 ± 0.40**	**<0.01**
**Others**										
**CD133 (*n* ≥ 7)**	0.95 ± 0.36	1.16 ± 0.95	0.58	**1.43 ± 0.39**	**<0.05**	1.02 ± 0.22	**1.46 ± 0.41**	**<0.05**	**0.67 ± 0.14**	**<0.01**
**Myc (*n* ≥ 9)**	1.01 ± 0.18	**0.49 ± 0.21**	**<0.0001**	**0.56 ± 0.20**	**<0.01**	1.01 ± 0.18	1.46 ± 0.43	0.06	**0.71 ± 0.30**	**<0.01**
**CyclinD1 (*n* ≥ 8)**	1.02 ± 0.19	**0.62 ± 0.18**	**<0.001**	**0.50 ± 0.22**	**<0.001**	1.00 ± 0.06	**1.23 ± 0.26**	**<0.05**	1.00 ± 0.29	0.96

Gene expression data are displayed as fold-change value (2^–ΔΔ*Ct*^) relative to vector control cells (mean ± SD). Statistically significant data are displayed in bold typing. *HS2ST3 is not expressed in MCF-7 and MDA-MB-231 cells. – denotes absence of expression. +++, denotes high expression due to stable transfection. **, Notch4 expression data were at the limit of detection (Ct values > 32). n.d., not determined. n.a., not applicable.

Next, we analyzed the activity of the enzyme ALDH1, another BCSC marker, by flow cytometry (ALDEFLUOR^TM^ assay). In MDA-MB-231 cells, ALDH1 activity increased from 2.88% (±0.66%) in the control transfected cells to 9.99% (±0.76%) after overexpression of HS2ST1, and 5.36% (±0.33%) in the HS3ST2 overexpressing cells (Figure 1B). HS2ST1 overexpression in the MCF-7 cells had the inverse effect, decreasing the activity of ALDH from 4.42% (±0.34%) in the control transfected cells to 0.91% (±0.10%) after its overexpression (Figure 1B).

### HS2ST1 and HS3ST2 Overexpression Modifies Colony Formation, and Number and Size of MDA-MB-231 and MCF-7 Tumor Spheres

**FIGURE 2:**
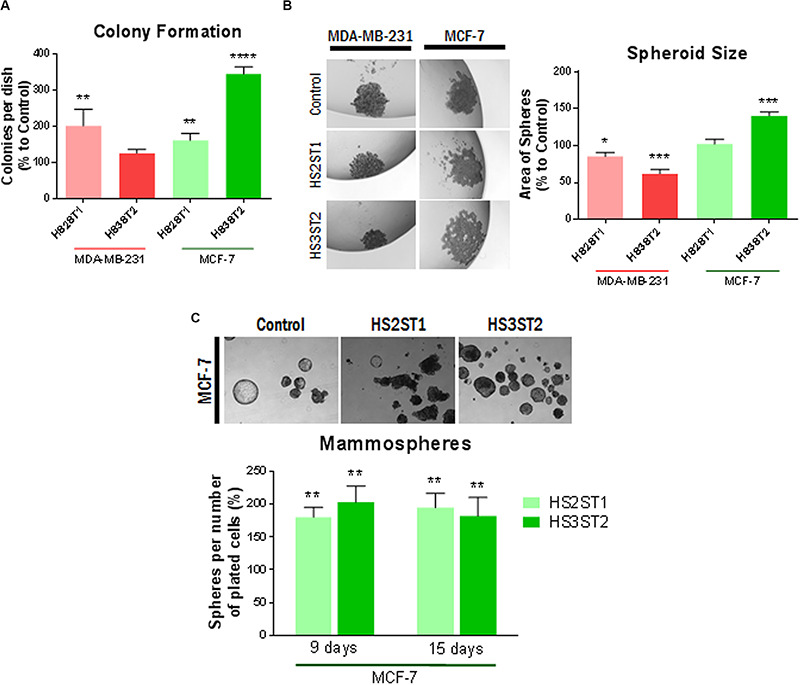
Functional assays for the CSC-phenotype. MDA-MB-231 and MCF-7 cell lines modified to overexpress HS2ST1 and HS3ST2 and control cells were submitted to a series of functional assays to investigate the acquisition of a CSC-phenotype. From top to bottom: **(A, Colony Formation)** Percentage of colonies relative to control (100%) present in each well after 14 days. **(B, Spheroid formation)** Examples of spheres found in the drops (left). Area of the spheres relative to the vector controls (100%) present in each drop after 1 week (right). **(C, Mammosphere assay)** Examples of mammospheres generated after 15 days (top). Percentage of mammospheres formed per number of plated cells relative to control (100%) in the MCF-7 cell line after 9 or 15 days. Data represent mean ± SD, *n* = 3. *P < 0.05, **P < 0.01, ***P < 0.001, ****P < 0.0001.

One of the main features related to the high tumorigenicity and self-renewal capacity of CSCs is the ability to form colonies *in vitro* after being seeded in low concentrations on cell culture plates (Yang et al., 2017). Overexpression of HS2ST1 significantly increased the number of colonies per dish by 101.86% in MDA-MB-231 (Figure 2A) and 61.93% in MCF-7 cells. HS3ST2 overexpression strongly increased the number of colonies per dish by 245.6% only in the MCF-7 cells (Figure 2A).

**FIGURE 3:**
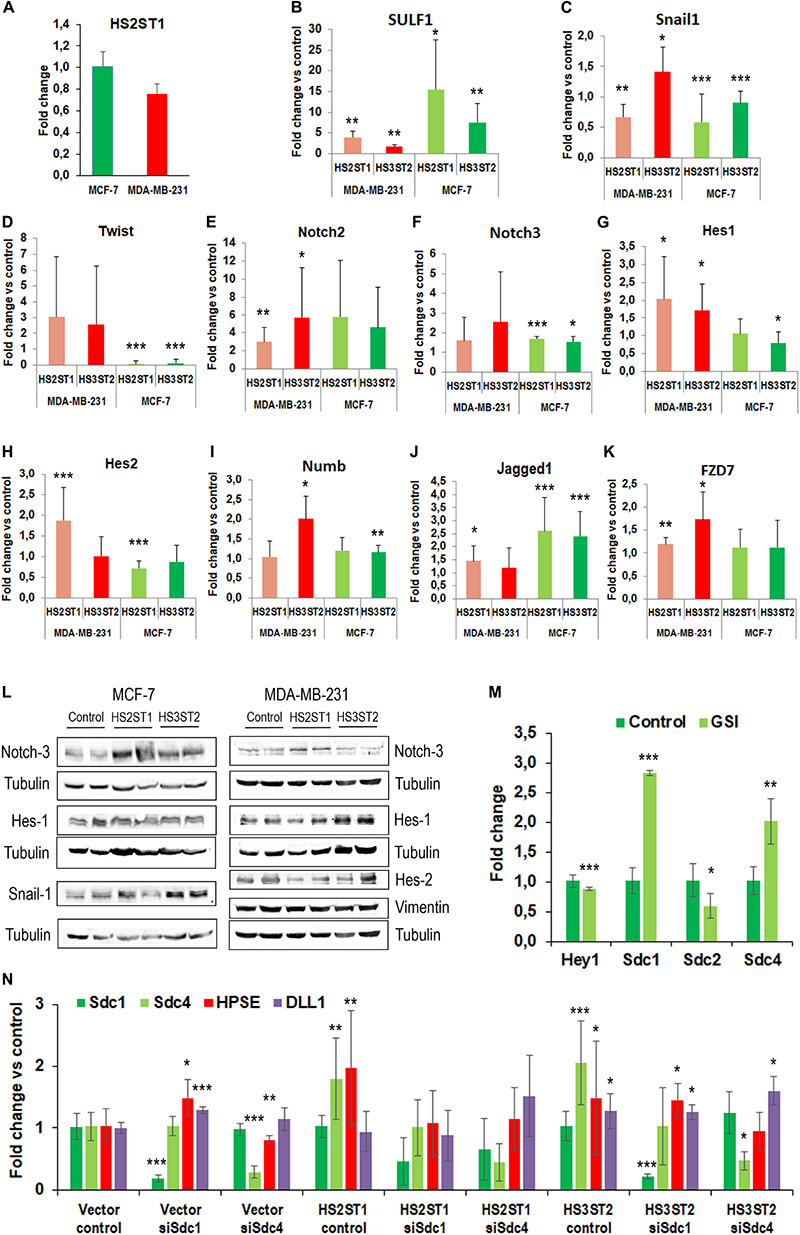
Impact of HS sulfotransferases on HS- and stemness-associated gene expression. **(A–K)** Total RNA was isolated from the cells, reverse transcribed into cDNA and subjected into quantitative real-time PCR for analysis of Notch pathway related genes. Data represent mean ± SD from at least three independent experiments (see Table 1 for **N**). *P < 0.05, **P < 0.001, ***P < 0.0001, **L**) Western blot analysis of stemness-associated factors in HS2ST1- and HS3ST2 overexpressing cells compared to controls. Loading control = tubulin. Representative data from at least 4 biological replicates. **(M)** Impact of notch pathway inhibition with gamma-secretase inhibitor (GSI) on Hey1 and Sdc-family members in MCF-7 cells. qPCR analysis as described in **(A–K)**. *N* ≥ 3. **(F)** Impact of Sdc-1 and Sdc-4 knockdown on altered gene expression in HS-sulfotransferase overexpressing MCF-7 cells. Data are shown relative to vector control cells as described in **(A–K)**. *N* ≥ 3.

Three-dimensional cell culture models mimic important features of the tumor, such as spatial organization, cell–cell interaction, differentiation, tumor growth and hypoxia (Djomehri et al., 2019). One important scaffold-free liquid-based system to form such spheroids is the hanging drop technique. In this assay, MDA-MB-231 cells could not form compact spheres but formed compact aggregates of cells (Figure 2B). The overexpression of HS2ST1 decreased the average size of the aggregates by 14.71% (±3.2%) and the overexpression of HS3ST2 decreased its size by 37.93% (±3.34%), compared to control. MCF-7 cells, on the other hand, generated larger spheres after overexpression of HS3ST2, an increase of 40.48% (±2.85%) on the average size compared to control (Figure 2B).

Since the MCF-7 cells seemed to form more compact and bigger spheres by the hanging drop method, we further analyzed the sphere formation capacity in non-adherent culture conditions, which promotes sphere formation from single cells and allows us to quantify the number of generated spheres. After 9 and 15 days of non-adherent culture, sphere formation was around 75% more efficient on both transfected cells. This result corroborates the hypothesis that HS2ST1 and HS3ST2 overexpression modifies and possibly enhances the stem-cell phenotype of these cells (Figure 2C).

### The Overexpression of HS2ST1 and HS3ST2 Regulates the Expression of Factors Related to EMT and Stem Cell Function

Recent developments in the field demonstrate that many tumor cells undergo epithelial-to-mesenchymal transition (EMT) and the reverse process (MET) in order to acquire the CSC phenotype (Zhang and Weinberg, 2018). This prompted us to analyze the expression of EMT markers in HS2ST1 and HS3ST2 overexpressing MDA-MB-231 and MCF-7 cells (Table 1). Moreover, as a link between HS2ST1 and the expression of PGs of the syndecan-family has recently been demonstrated (Kang et al., 2020), we investigated the expression of all family members, of the functionally linked HS degrading enzyme heparanase (HPSE) and of the HS sulfate editing enzymes SULF1 and SULF2 by qPCR. HS2ST1 overexpression levels were comparable in transfected MCF-7 and MDA-MB-231 cells (Figure 3A). Regarding further HS-related gene expression, we noted a significant upregulation of SULF1 in all sulfotransferase-expressing cells, whereas SULF2 was downregulated in HS3ST2 overexpressing MCF-7 cells (Table 1 and Figure 3B). HPSE and SDC4 were significantly upregulated in MCF-7 cells overexpressing HS2ST1 and HS3ST2. In contrast, HPSE was significantly downregulated, and SDC2 upregulated in HS2ST1-transfected MDA-MB-231 cell, indicating context-dependent effects (Figure 3 and Table 1). Regarding EMT-related factors, in MCF-7 cells, overexpression of HS2ST1 and HS3ST2 reduced the gene expression of Vimentin, a marker of mesenchymal cells, and of the EMT-inducing transcription factors Snail1, Twist and Snail 2 (only HS2ST1), compared to the vector control cells. *E*-cadherin gene expression was reduced, while β-catenin was upregulated (Table 1 and Figures 3C,D). In MDA-MB-231 cells, we observed a significant reduction of Vimentin and ZEB1 mRNA expression after HS3ST2 overexpression, and of Snail1 and Snail2 after HS2ST1 overexpression. In contrast, these two factors were significantly upregulated in HS3ST2-overexpressing MDA-MB-231 cells. While the expression of *E*-cadherin was not significantly affected, *N*-cadherin was moderately upregulated upon HS2ST1 upregulation in this cell line (Table 1). Overall, our data indicate a general trend for downregulation of mesenchymal markers upon sulfotransferase overexpression, with context-dependent cell-type and sulfotransferase-specific effects, as also noted for the transcription factor Myc, the stem cell marker CD133 and the proliferation marker CyclinD1 (Table 1).

The Notch pathway plays an important role in the activation and maintenance of CSCs (Hovinga et al., 2010; Chen et al., 2014; Takebe et al., 2015). Notably, Syndecan HSPGs modulate notch signaling, suggesting an impact of HS on this pathway (Ibrahim et al., 2017; Vitale et al., 2019). In line with this hypothesis, the MDA-MB-231 cell line showed an apparent increase in the activation of the Notch pathway after HS sulfotransferase upregulation (Figure 3). In HS2ST1 overexpressing MDA-MB-231 cells, an increase in the expression of Notch2, of the transcriptional regulators HES1 and HES2, markers of activation of this pathway, and of the notch ligands DLL1 and JAG1 was observed, whereas the notch modulator NUMB was also upregulated. Overexpression of HS3ST2 was associated with significant upregulation of Notch2, Hes1, and Hey1, another notch activation marker (Table 1 and Figures 3E–J). HS2ST1 overexpressing MCF-7 cells showed upregulation of Notch-3, Hey1, Hey2, and Jag1 and downregulation of Hes2, whereas HS3ST2 overexpression resulted in upregulation of Notch-3, NUMB, DLL1, Hey2 and JAG1, and downregulation of DLL4 and Hes1 (Figures 3E–J and Table 1). qPCR analysis of Wnt pathway-related genes revealed a downregulation of the transcription factor TCF7L1 in HS –sulfotransferase overexpressing MCF-7 cells, whereas the Wnt receptor FZD7 was upregulated, and Wnt5a was downregulated in HS sulfotransferase overexpressing MDA-MB-231 cells (Table 1 and Figure 3K). Wnt1 was upregulated in HS3St1 overexpressing MDA-MB-231 only (Table 1). We next confirmed selected results at the protein level (Figure 3L). Western blot analysis revealed upregulation of Notch3 in Sulfotransferase overexpressing MCF-7 cells, and in HS2ST1 overexpressing MDA-MB-231 cells. Results for Hes1 were ambiguous, indicating a slight upregulation in HS3ST2 expressing MDA-MB-231 cells. Hes2 was downregulated in HS2ST1 overexpressing MDA-MB-231 cells, whereas Vimentin protein expression (undetectable in epithelial MCF-7 cells) was largely unaltered. In MCF-7 cells, HS3ST2 overexpression resulted in an upregulation of Snail1 protein (Figure 3L).

### The Syndecan Family of HSPGs and the Notch Pathway Are Part of the Regulatory Circuit of HS Sulfotransferases

As our gene expression analysis had indicated a complex impact of altered HS sulfotransferase expression on stemness-related signaling pathways and on members of the syndecan family, we further explored the interdependence of these pathways in inhibitor studies. Application of gamma secretase inhibitor (GSI), which inhibits both activation of the notch pathway and shedding of Sdc-1 (Pasqualon et al., 2015; Ramirez Williams et al., 2019) resulted in a modest, but significant inhibition of Hey1 and Sdc-2 expression, whereas Sdc-1 and Sdc-4 were strongly upregulated in MCF-7 cells (Figure 3M). As HS2ST1-dependent modification of Sdc-1 has recently been linked to breast cancer pathogenetic properties (Kang et al., 2020), we employed an siRNA depletion approach to downregulate the expression of Sdc-1 and Sdc-4 in sulfotransferase overexpressing MCF-7 cells, followed by qPCR analysis of HSPE, Sdc-1, Sdc4 and the notch ligand DLL1. Sdc-1 knockdown resulted in upregulation of HPSE and DLL1 in control cells, and abolished HS2ST1-dependent upregulation of Sdc-4 and HPSE, and HS3ST2 dependent upregulation of Sdc-4 (Figure 3N). Sdc-4 siRNA knockdown was associated with HSPE downregulation in control cells, and abolished HS2ST1 and HS3ST2-dependent HPSE upregulation in MCF-7 cells. Overall, these data provide evidence for a complex regulatory interplay of HS sulfotransferases, syndecans and the notch signaling pathway.

## Discussion

Studying HS sulfotransferases is a promising tool for understanding the biological functions of these enzymes on the tumor cell phenotype. Previous studies demonstrated an important role of HS3ST2 and HS2ST1 and the associated changes in HS structure in modulating receptor tyrosine kinase dependent signaling and breast cancer cell invasion, proliferation and senescence (Vijaya Kumar et al., 2014, 2020; Kang et al., 2020). However, the functional impact on CSCs was unknown. Here, we demonstrate an impact of altered HS2ST1 and HS3ST2 expression on the CSC phenotype, which is associated with complex expression changes in the stemness-associated Notch and Wnt signaling pathways, and with altered expression of proteoglycans of the syndecan family. Similar to our previous work on HS3ST2 (Vijaya Kumar et al., 2014), we observed both common and context-dependent effects of altered HS sulfotransferase expression. In the present study, colony and mammosphere formation – two important functional readouts of CSCs - were consistently upregulated in both model cell lines upon overexpression of both sulfotransferases, indicating a stemness-promoting function of these enzymes. At the molecular level, our data point at an upregulation of several components of the notch signaling pathway, which we previously linked to Sdc-1 function in breast and colon cancer (Ibrahim et al., 2017; Katakam et al., 2020a). For example, Sdc-1 expression in inflammatory breast cancer is correlated with CD44, Notch-1, and Notch-3 expression, and siRNA knockdown of Sdc-1 results in a weaker CSC phenotype and reduced expression of Notch-1-4 and Hey1 in inflammatory breast cancer cells (Ibrahim et al., 2017). Our data are in line with these findings, demonstrating that altered HS structure is linked to the CSC phenotype as well as altered expression of syndecans and notch constituents. While our GSI inhibitor studies demonstrate that the notch pathway has a regulatory impact on syndecan expression, our Sdc siRNA data reveal context-dependent effects. Knockdown of Sdc-4 largely affected basal and HS-sulfotransferase dependent HPSE expression, whereas Sdc-1 knockdown affected HPSE, Sdc-4 and DLL1. The underlying mechanisms are apparently complex and require further study. Obvious mechanisms include altered receptor tyrosine kinase signaling conform with the coreceptor concept of HSPGs, as demonstrated for the MAPK pathway in HS2ST1 and HS3ST2 overexpressing cells (Vijaya Kumar et al., 2014, 2020), and altered signaling via the Wnt pathway, as exemplified by altered expression of the Wnt-dependent transcription factor TCF4 for both sulfotransferases, and by the Sdc-1 and Wnt-dependent modulation of a colon cancer stem cell phenotype (Katakam et al., 2020b). Our finding of a upregulated expression of the Wnt receptor FZD7 in HS sulfotransferase overexpressing MDA-MB-231 cells may be mechanistically relevant, as this receptor acts along with Sdc-4 during foregut progenitor development (Zhang et al., 2016). Moreover, altered HS sulfation patterns, including upregulation of 2-O- and 6-O-sulfation, are linked to myoblast cell fate and FGF2 signaling (Ghadiali et al., 2017). However, more complex regulatory mechanisms may occur at the level of cell surface availability of signaling receptors and co-receptors. For example, both the shedding of Sdc-1 and activation of notch can be mediated by gamma-secretase (Pasqualon et al., 2015), and Sdc-1 shedding is regulated by HPSE (Rangarajan et al., 2020), which is expressed in a HS-sulfotransferase-dependent manner according to our study. Finally, HS2ST1-modified Sdc-1 was shown to prevent cellular senescence through the regulation of FGFR1 endocytosis (Kang et al., 2020), and similar mechanisms could play a role in the HS2ST1-dependent CSC phenotype. With respect to HS3ST2, our observation of an impact on the CSC phenotype are in line with studies that showed an important function of HS 3-O-sulfation in the differentiation of murine embryonic stem cells, as demonstrated by upregulation of 3-O-sulfated HS structures during critical stages of differentiation, and by functional knockdown studies on another 3-O-Sulfotransferase, 3OST-5 (Hirano et al., 2013). Along with previous reports on a potential role of 3-O-Sulfation in notch signaling in the fruit fly (Kamimura et al., 2004), our data extend this concept to the field of CSC research. As an interesting finding, we have observed upregulation of the HS editing enzyme SULF1 in both HS3ST2 and HS2ST1 overexpressing MDA-MB-231 cells. While this observation (along with upregulation of HPSE) may be a cellular attempt to compensate for the alterations in HS structure exerted by sulfotransferase dysregulation, it may be relevant in the context of stem cell function as well: For example, Sulf1 is required for the termination of Drosophila intestinal stem cell division during regeneration (Takemura and Nakato, 2017), and regulates Wnt signaling in the context of myoblast fusion (Tran et al., 2012).

Apart from notch and Wnt signaling, our study has revealed an impact on the expression of EMT markers. While we previously demonstrated upregulation of *E*-cadherin protein in HS2ST1 and HS3ST2 overexpressing MDA-MB-231 and MCF-7 cells (Vijaya Kumar et al., 2014, 2020), the demonstration of downregulated mesenchymal factors is a new finding of this study. Snail1 and Snail2 are implicated in EMT via the upregulation of mesenchymal markers such as vimentin and suppression of epithelial markers such as *E*-cadherin. The link between EMT and the CSC phenotype may serve to explain differences in the impact of HS2ST1 and HS3ST2 on the different model cell lines of this study, as MCF-7 cells show an epithelial phenotype, whereas MDA-MB-231 cells have mesenchymal properties. Notably, mesenchymal (CD44^+^/CD24^–^) and epithelial (ALDH^+^) CSCs are two distinct populations with different functionalities (Liu et al., 2014). Overexpression of HS2ST1 and HS3ST2 decreased the stemness-associated CD44^+^/CD24^–^ phenotype only in the MDA-MB-231 cell line. CD44 is associated with a mesenchymal phenotype, while CD24 is associated with an epithelial phenotype in breast cancer (Jaggupilli and Elkord, 2012). From a phenotypic point of view, these changes may mean a change in the epithelial or mesenchymal phenotype of these cells, possibly leading to the acquisition of a CSC phenotype as well. In MDA-MB-231 cells a decrease in the CD44^+^/CD24^–^ phenotype could be transitioning to an epithelial-like state, which is corroborated by a greater ALDH1 activity. In MCF-7 cells, there is less activity of ALDH1 upon HS2ST1 overexpression, which could possibly lead to a mesenchymal-like phenotype (see Ibrahim et al., 2013, for discussion). Considering the analysis of CD44, CD24, and ALDH, it seems that overexpression of HS3ST2 triggered antagonistic results compared to those obtained by HS2ST1 overexpression in both MDA-MB-231 and MCF-7 cells. We can only speculate that the HS pattern generated by HS3ST2 overexpression may be capable of activating or inactivating ligands that are not affected in the same manner by HS2ST1-modified HS, and vice versa. Famous examples are specific structural requirements for HS interactions, such as the antithrombin binding motif of the FGF2 binding sequence (Karamanos et al., 2018). For example, we have demonstrated that HS2ST1 upregulation in our cell lines results in reduced surface binding of FGF2 (Vijaya Kumar et al., 2020). The differential regulation of distinct components of the Wnt and notch signaling pathway in our cells supports this view (Table 1). Furthermore, context-dependent effects of HS in different cell types can be explained by the fact that not all ligands and receptors that are influenced by HS are expressed by all cells. For example, Wnt1 was upregulated in HS3ST2 overexpressing MDA-MB-231 cells, while this factor was not expressed in MCF-7 cells and could therefore not be affected by this HS modification.

Regarding our functional readouts, the origin of our cells (epithelial vs. mesenchymal) may have influenced the impact of HS-dependent changes in EMT markers, resulting in the formation of smaller and more compact aggregates in the case of sulfotransferase overexpressing MDA-MB-231 cells, which may have shifted toward a more epithelial-like phenotype, as discussed previously (Figure 2B). Some caveats are associated with our research communication. Several findings of our study rely on mRNA expression data and require further validation at the protein and functional level. While we could, e.g., confirm HS-sulfotransferase-dependent upregulation of Notch-3 in both cell lines, and of Hes-1 in HS3ST2-expressing MDA-MB-231 cells, other factors were either less consistently regulated at the mRNA and protein level, or have not been confirmed, yet. In addition, not all factors within a given pathway were consistently regulated in the same direction, and some factors were only moderately altered, requiring more study. Our GSI inhibitor and siRNA studies demonstrate that the signaling pathways and compounds involved are highly interdependent and subject to potential compensatory mechanisms. While this observation impedes a straightforward and simple mechanistic explanation, it reflects the complexity of HS-dependent processes, which affect signaling via numerous pathways, and additional cellular functions beyond classical signaling, such as endocytosis (affecting receptor downregulation), proteolysis and cell-matrix interactions (Karamanos et al., 2018). Finally, while sulfotransferase overexpression promoted functional stem cell properties, it is likely that not only the CSC population, but also the overall tumor cell population was affected by alterations in HS. Possibly a selected analysis of sorted CSCs could lead to an enhancement of the observed changes, as previously demonstrated for the impact of Sdc-1 knockdown on the colon CSC phenotype (Katakam et al., 2020b).

Taken together, HS2ST1 and HS3ST2 partially had a differential impact on the CSC phenotype of representative triple-negative and hormone-receptor positive breast cancer cell lines. This finding may reflect differences in HS-dependent signaling pathways, as previously shown for the invasion phenotype of HS3ST2 overexpressing cells (Vijaya Kumar et al., 2014). Our results furthermore show that the overexpression of HS2ST1 and HS3ST2 significantly alters several CSC-related characteristics in breast cancer cells in general, which is worthy of future evaluation in more complex *in vivo* systems. Finally, our data open a perspective for manipulating the CSC phenotype with drugs modulating HS either in a general way, or in a sequence-specific manner (Zubkova et al., 2018; Vitale et al., 2019; Espinoza-Sánchez and Götte, 2020).

## Data Availability Statement

All datasets generated for this study are included in the article/Supplementary Material.

## Author Contributions

FT, AV, and SK performed the major part of the experiments and analyzed the data. CC, PP, and RR performed the mammosphere assays, analyzed the data, and provided the expertise on CSC analysis. MoG performed the siRNA experiments and qPCRs. FT drafted the figures and wrote the draft of the manuscript. MaG, MP, and BG supervised the data. LK provided the general support, co-supervised the MoG, and was involved in the data interpretation. MaG conceived and coordinated the study and generated the figures and tables of the revised manuscript. All authors revised and approved the final draft.

## Conflict of Interest

The authors declare that the research was conducted in the absence of any commercial or financial relationships that could be construed as a potential conflict of interest.

## Abbreviations

ALDH: aldehyde dehydrogenase
BCSC: breast cancer stem cell
CSC: cancer stem cell
ECM: extracellular matrix
ER: estrogen receptor
GAG: glycosaminoglycan
HS: heparan sulfate
MFI: mean fluorescence intensity
NDST: *N*-deacetylase /*N*-sulfotransferase
PBS: phosphate-buffered saline
PR: progesterone receptor.
