# Supplementary material for: The Heparan Sulfate Sulfotransferases HS2ST1 and HS3ST2 Are Novel Regulators of Breast Cancer Stem-Cell Properties
Source: Front Cell Dev Biol. 2020 Sep 25;8:559554. doi: 10.3389/fcell.2020.559554 (PMC7546021; doi:10.3389/fcell.2020.559554)
Supplement: Supplementary file 1 [file Table_1.docx]

**Supplemetary Table I.** PCR primers used in Teixeira et al.

| **SYBR Green primer** | **Primer sequence (5’-3’)** |
| --- | --- |
|  |  |
| ACTB1 (beta-Actin) fwd | CATGTACGTTGCTATCCAGGC |
| ACTB1 (beta-Actin) rev | CTCCTTAATGTCACGCACGAT |
|  |  |
| CCND1 fwd | ACGAAGGTCTGCGCGTGTT |
| CCND1 rev | CCGCTGGCCATGAACTACCT |
|  |  |
| CD133 fwd | TCAAAGATTGGCCATGTTCCAC |
| CD133 rev | TGTCAGATGGAGTTACGCAGGTT |
|  |  |
| CDH1 fwd | CAAAGCCCAGAATCCCCAAG |
| CDH1 rev | CACACCTGGAATTGGGCAAA |
|  |  |
| CDH2 (N-Cadherin) fwd | TTCTGACAACAGCTTTGCCTCTG |
| CDH2 (N-Cadherin) rev | TTTATTCAGAACGCTGGGGTCA |
|  |  |
| CTNNB1 (beta-catenin) fwd | TTTGGACAGTTTACCAGTTGCCTTT |
| CTNNB1 (beta-catenin) rev | CTCTTGAAGCATCGTATCACAGCA |
|  |  |
| HEY2 fwd | TTGCCCATGCCTAAACTAGTGC |
| HEY2 rev | TCTCACGTGCTTGATTTCAGCATA |
|  |  |
| MYC fwd | CGGAACTCTTGTGCGTAAGGAA |
| MYC rev | GCCAAGGTTGTGAGGTTGCAT |
|  |  |
| NOTCH 3 fwd | TGTGCAAATGGAGGTCGTT |
| NOTCH 3 rev | CCTGAGTGACAGGGGTCCT |
|  |  |
| SDC1 fwd | AGGACGAAGGCAGCTACTCCT |
| SDC1 rev | TTTGGTGGGCTTCTGGTAGG |
|  |  |
| SDC2 fwd | GCTGATGAGGATGTAGAGAG |
| SDC2 rev | CACTGGATGGTTTGCGTTC |
|  |  |
| SDC3 fwd | TGCCTCAGAAGAGTATCCTG |
| SDC3 rev | CTTGTCAGGCTTCTGGTATG |
|  |  |
| SDC4 fwd | TGTCCAACAAGGTGTCAATG |
| SDC4 rev | GTTTCTTGCCCAGGTCATAG |
|  |  |
| SNAI1 fwd | CGAGCCCAGGCAGCTATTTC |
| SNAI1 rev | CCCGACAAGTGACAGCCATT |
|  |  |
| SNAIL2 fwd | ATCTGCCAGACGCGAACTCA |
| SNAIL2 rev | GGCAACCAGACAACCGACAT |
|  |  |
| TWIST fwd | GCGGCCAGGTACATCGACTT |
| TWIST rev | TGCAGCTTGCCATCTTGGAG |
|  |  |
| TCF7L1 fwd | AAGGTGCCTGCCACTTCCTC |
| TCF7L1 rev | CCTGCCACTCTGGGATTGTG |
|  |  |
| Vimentin fwd | TCAGCATCACGATGACCTTGAA |
| Vimentin rev | CTGCAGAAAGGCACTTGAAAGC |
|  |  |
| Wnt3a fwd | AGGTCCCACAGCCCTGAGAT |
| Wnt3a rev | TCCAGGAAAGCGGACCATTT |
|  |  |
| Wnt5a fwd | TCGTTAGCAGCATCAGTCCACA |
| Wnt5a rev | GACCTGTGCCTTCGTGCCTA |
|  |  |
| ZEB1 fwd | CAGCCCTGCAGTCCAAGAAC |
| ZEB1 rev | TTGTCTTTCATCCTGATTTCCATTT |
|  |  |
| ZEB2 fwd | TGGGCTAGTAGGCTGTGTCCA |
| ZEB2 rev | TCATCTTCAACCCTGAAACAGAGG |
|  |  |
| **Tag Man Probes** | **ABI Probe ID*** |
|  |  |
| 18S rRNA | Hs99999901_s1 |
| DLL1 | hs 00194509 m1 |
| DLL 3 | hs 01085096 m1 |
| DLL 4 | hs 00184092 m1 |
| HES 1 | hs 00172878_m1 |
| HES 2 | hs 00219505_m1 |
| HEY 1 | hs 00232618 m1 |
| HPSE | hs 00180737 m1 |
| HS2ST1 | hs_00202138_m1 |
| HS3ST2 | hs_00428644_m1 |
| JAG 1 | hs 01070032_m1 |
| NOTCH 1 | hs 00413187 m1 |
| NOTCH 2 | hs 01050719 m1 |
| NOTCH 4 | hs 00965897 m1 |
| NUMB | hs 00377772_m1 |
| SFRP 1 | hs 006110060 m1 |
| SULF 1 | hs 00290918 m1 |
| SULF 2 | hs 00378697 m1 |
| WNT 1 | hs 00180529 m1 |
| *https://www.thermofisher.com/de/en/home/life-science/pcr/real-time-pcr/real-time-pcr-assays.html | |
