# Supplementary material for: The Heparan Sulfate Sulfotransferases HS2ST1 and HS3ST2 Are Novel Regulators of Breast Cancer Stem-Cell Properties
Source: Front Cell Dev Biol. 2020 Sep 25;8:559554. doi: 10.3389/fcell.2020.559554 (PMC7546021; doi:10.3389/fcell.2020.559554)
Supplement: Supplementary file 2 [file Table_2.docx]

**Supplementary Table II:** List of antibodies used in Teixeira et al.

| **Antibody and dilution** | **manufacturer** |
| --- | --- |
| anti-human-Snail-1, C15D1, #3879s, Rabbit, 1:1000 | Cell Signaling Technologies, Frankfurt a.M, Germany |
| anti-human-Notch-3, C15D1, #5276s, Rabbit, 1:1000 | Cell Signaling Technologies, Frankfurt a.M, Germany |
| anti-human-α-Tubulin, clone B-5-1-2, Mouse, 1:1000 | Sigma-Aldrich, Steinheim, Germany |
| anti-human Hes1, )sc51477(, Mouse, 1:1000 | Santa Cruz Biotechnology, Santa Cruz, USA |
| anti-human Hes2, )H-8(, Mouse, 1:1000 | Santa Cruz Biotechnology, Santa Cruz, USA |
| anti-human Vimentin, )H-84(, Rabbit, 1:1000 | Santa Cruz Biotechnology, Santa Cruz, USA |
| anti-mouse-IgG, H&L-Chain, HRP-Conj., Goat, 1:10000 | Merck KGaA, Darmstadt, Germany |
| anti-rabbit-IgG, H&L-Chain, HRP-Conj., Goat, 1:2000 | Merck KGaA, Darmstadt, Germany |
